# Transcriptionally active nasopharyngeal commensals and opportunistic microbial dynamics define mild symptoms in the COVID 19 vaccination breakthroughs

**DOI:** 10.1371/journal.ppat.1011160

**Published:** 2023-02-17

**Authors:** Priti Devi, Pallawi Kumari, Aanchal Yadav, Bansidhar Tarai, Sandeep Budhiraja, Uzma Shamim, Rajesh Pandey

**Affiliations:** 1 Division of Immunology and Infectious Disease Biology, INtegrative GENomics of HOst-PathogEn (INGEN-HOPE) laboratory, CSIR-Institute of Genomics and Integrative Biology (CSIR-IGIB), Delhi, India; 2 Academy of Scientific and Innovative Research (AcSIR), Ghaziabad, India; 3 Max Super Speciality Hospital (A Unit of Devki Devi Foundation), Max Healthcare, Delhi, India; Universite Paris Descartes Faculte de Medecine, FRANCE

## Abstract

The development of COVID 19 vaccines as an effort to mitigate the outbreak, has saved millions of lives globally. However, vaccination breakthroughs have continuously challenged the vaccines’ effectiveness and provided incentives to explore facets holding potential to alter vaccination-induced immunity and protection from subsequent infection, especially VOCs (Variants Of Concern). We explored the functional dynamics of nasopharyngeal transcriptionally active microbes (TAMs) between vaccination breakthroughs and unvaccinated SARS-CoV-2 infected individuals. Microbial taxonomic communities were differentially altered with skewed enrichment of bacterial class/genera of *Firmicutes* and *Gammaproteobacteria* with grossly reduced phylum *Bacteroidetes* in vaccination breakthrough individuals. The *Bacillus* genus was abundant in *Firmicutes* in vaccination breakthrough whereas *Prevotella* among *Bacteroides* dominated the unvaccinated. Also, *Pseudomonas* and *Salmonella* of *Gammaproteobacteria* were overrepresented in vaccination breakthrough, whilst unvaccinated showed presence of several genera, *Achromobacter*, *Bordetella*, *Burkholderia*, *Neisseria*, *Hemophilus*, *Salmonella* and *Pseudomonas*, belonging to Proteobacteria. At species level, the microbiota of vaccination breakthrough exhibited relatively higher abundance of unique commensals, in comparison to potential opportunistic microbes enrichment in unvaccinated patients’ microbiota. Functional metabolic pathways like amino acid biosynthesis, sulphate assimilation, fatty acid and beta oxidation, associated with generation of SCFAs (short chain fatty acids), were enriched in vaccination breakthroughs. Majorly, metabolic pathways of LCFAs biosynthesis (long chain fatty acids; oleate, dodecenoate, palmitoleate, gondoate) were found associated with the unvaccinated. Our research highlights that vaccination decreases the microbial diversity in terms of depleting opportunistic pathogens and increasing the preponderance of commensals with respect to unvaccinated patients. Metabolic pathway analysis substantiates the shift in diversity to functionally modulate immune response generation, which may be related to mild clinical manifestations and faster recovery times during vaccination breakthroughs.

## Author summary

First reported on 31 December, 2019, in Wuhan, the COVID 19 pandemic, brought on by the SARS-CoV-2 infection, resulted in innumerable human lives lost, and still continues to cause time-to-time outbreaks. Millions of lives have been saved worldwide thanks to increased vaccination coverage, but there exist numerous vaccine breakthrough infections. Here, we employed the RNA-Seq approach to identify the distinct transcriptionally active microbial signatures between the vaccination breakthrough and unvaccinated SARS-CoV-2 infected patients. The taxonomic profile and the functional pathways from the metatranscriptomic data points towards increased abundance of commensal and opportunistic microbial species in vaccination breakthrough and unvaccinated groups respectively, supported by differentially enriched pathways between the two groups. These data suggest that vaccination enhances the prevalence of commensals and depletes opportunistic bacteria in comparison to unvaccinated patients. *P*. *alcaliphila* seems to be one of the potential commensals dominating the nasopharyngeal microbiota of vaccination breakthroughs. Together these data underscore the role of microbes in modulating disease trajectory and suggest contribution to faster recovery and milder symptoms in the vaccination breakthrough cases signifying the protective role played by vaccines.

## Background

The World Health Organization (WHO) has characterized the infection by human pathogen, severe acute respiratory syndrome coronavirus 2 (SARS-CoV-2), as the main cause of the coronavirus disease 2019 (COVID 19) pandemic. As of 30^th^ August 2022, there have been 6.45 million deaths and 596 million confirmed cases of COVID 19 globally (https://covid19.who.int/). These patients exhibit mild to severe symptoms, with a multitude of them developing multiple organ failure and eventually leading to death. In light of this, multiple vaccines have been developed to effectively control the ongoing pandemic. COVID 19 vaccines, such as Covaxin (BBV152, whole inactivated virus-based vaccine) and Covishield (ChAdOx1 nCoV- 19 Recombinant Corona Virus Vaccine), majorly used in India, provide direct protection against infection and primarily prevent disease severity. Chandramani et al. demonstrated that fully vaccinated individuals from an eastern state of India were less likely to develop severe disease [1]. As much as vaccination reduces COVID 19 hospitalizations, there have been significant number of ’*breakthrough’* infections worldwide, including India, after double dose vaccination, thus allowing the chance of subsequent transmission [2] [3]. Consequently, it is imperative to understand why a subset of patients acquire infection/s after administration of vaccine doses? Equally important question is what leads to milder disease symptoms during vaccination breakthroughs? According to Amanatidou et al., breakthrough infections are caused by four major factors: vaccination parameters, viral traits, immune characteristics and host determinants [4]. While these are important aspects, human hosts are also home to a vast microbial community within us. The role of co-presence/co-infection of microbes have been recently highlighted as a plausible modulator of COVID 19 disease severity (mild, moderate and severe) and clinical outcome (recovered and mortality) [5] [6]. Whether the human resident nasopharyngeal microbes, especially transcriptionally active microbes (TAMs), play a role in vaccination breakthrough needs to be deciphered?

Although limited, it has been highlighted that vaccine-induced immune responses can be modulated by alterations in microbial composition [7]. Influenza vaccines have been studied primarily for their effect on nasal microbes and vice versa, while COVID 19 vaccines awaits focussed attention for their effect on active microbes in the human upper respiratory tract. Vaccines for influenza virus, whether live attenuated or killed, have been found to interact with the respiratory microbiota, with pneumonia associated taxa, *Streptococcus* being significantly enriched in the unvaccinated individuals [8]. Yet, another study discovered an underappreciated role of the microbiota in modulating trivalent inactivated influenza vaccine (TIV) immunity, wherein the contribution of the gut microbiota to TLR5-mediated augmentation of TIV immunity was highlighted [9]. On similar lines, Pneumococcal conjugate vaccines (PCVs) against Streptococcus pneumonia infection were shown to target the nasopharyngeal microbiome as well. PCV vaccination changed the carriage prevalence of several species, such as *S*. *aureus* and *H*. *influenza* [10]. These findings emphasize the necessity of identifying the factors, such as the use of antibiotics and probiotics, before or during vaccination, which may either modulate the host microbiota to boost vaccine efficacy or cause reinfections.

Nevertheless, there is a need to improve the efficiency of SARS-CoV-2 vaccine as well as associated adverse effects. It is worth mentioning that studies related to SARS-CoV-2 vaccination, especially vaccination breakthroughs, are scarce and not fully explored yet, with one prospective study identifying the correlation of gut microbiota composition with enhanced immune response and reduced adverse events following COVID 19 vaccines [11].

Thus, through the present cross-sectional study, we have explored and elucidated the functional role of transcriptionally active microbes (TAMs) using dual RNA-Sequencing (RNA-Seq) and metagenomic analysis of the nasopharyngeal microbiome in the SARS-CoV-2 infected individuals, between cohort of vaccination breakthrough and non-vaccinated infections. An integrated multilevel comparative analysis including taxonomic diversity and composition, microbial biomarker identification, relative abundance differences in the microbial species, metabolic pathways identification and its bacterial association, highlighted the functional role of the differentially abundant TAMs fostering favourable immune response. This helps to explain two important facets of faster recovery from the disease and milder symptoms in the vaccination breakthrough patients. Our work also builds on the earlier study from the lab wherein COVID 19 disease severity associated microbial signatures are validated in this cohort as well.

## Results

### Study design and clinical highlights of the patients

A total of 132 hospital admitted COVID 19 patients were included in the study. This includes, 1^st^ cohort of 58 hospital admitted COVID 19 patients who tested RT-PCR positive between Jan 2021-April 2021 were recruited for the vaccination breathrough study. These patients were also confirmed by whole genome sequencing of the SARS-CoV-2 using the nasopharyngeal RNA. The patients were categorized into two groups i.e., unvaccinated & SARS-CoV-2 infected (n = 29) and vaccination breakthrough (n = 29), wherein the vaccination breakthrough cases had received two doses of COVID 19 vaccine prior to the infection. The 2^nd^ cohort of 74 COVID 19 patients (from an earlier time frame, April-May 2020) with different disease severities (mild n = 24; moderate n = 36; severe n = 14) were also included for comparative microbial diversity analysis as well as focused validation of the microbial species associated with the disease severity [5].

**Fig 1:**
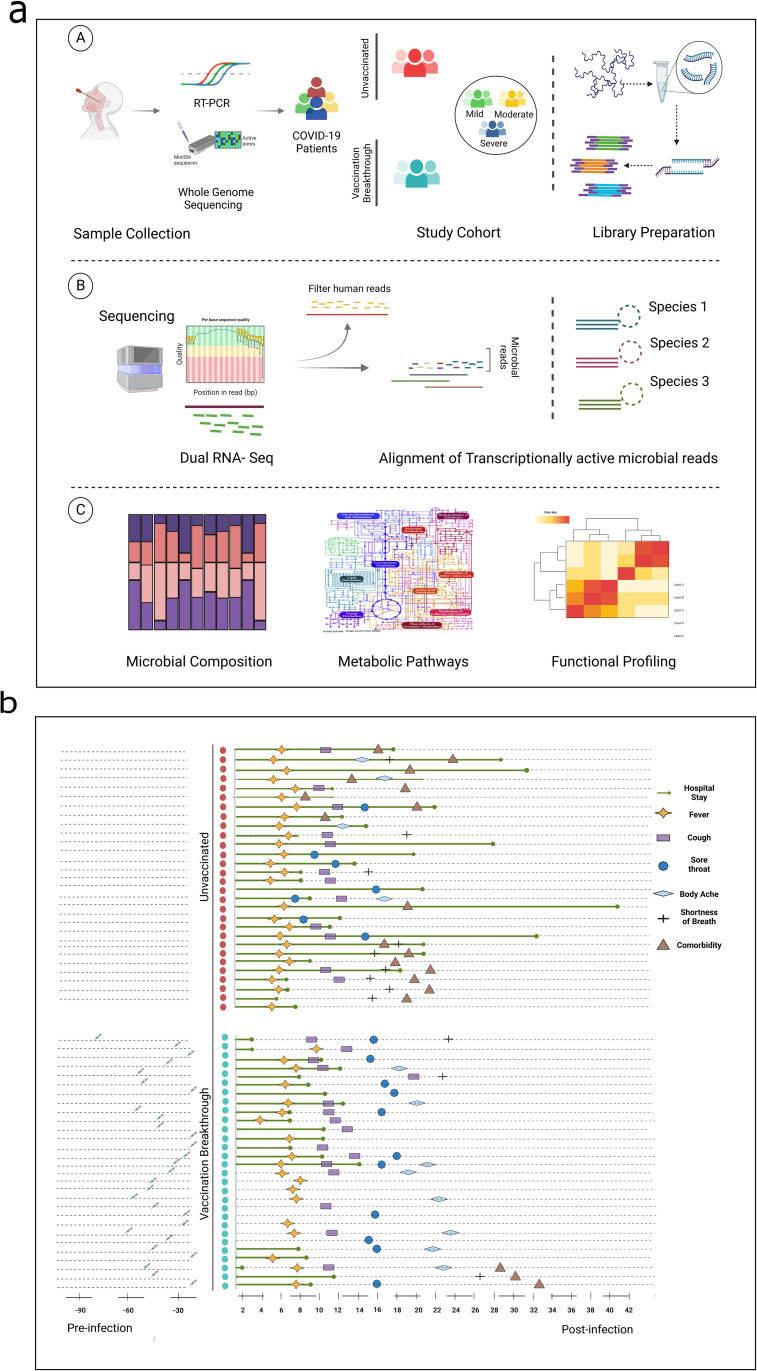
Summary of study cohort, dual RNA-seq, functional analysis, inference and clinical sample metadata. (a) Study design illustrating the patient cohorts, experimental workflow and functional interpretation of the analysis for the differentially abundant microbes. It also highlights the sample cohort of vaccination breakthrough/unvaccinated and the differential disease severity of mild, moderate and severe. (b) Individual plotting of clinical symptoms for unvaccinated and vaccination breakthrough patients with specific details of hospital stay, fever, cough, body ache, shortness of breath, comorbidities and vaccination status. Figure created with Biorender.com.

**Fig 1A** summarizes our study design demonstrating patient segregation, dual RNA-seq experimental workflow and the downstream analysis. Post dual RNA-sequencing from the nasopharyngeal RNA, the human reads were filtered out for the enrichment of the microbial reads that were obtained for each patient (Avg. reads ≈ 13529718) (**S1 File)**. For stringent analysis and inferences, two samples from the unvaccinated (UNinf) and one from the vaccination breakthrough (VB) were removed due to the relatively low microbial reads. Downstream functional analysis including microbial composition, species profiling and metabolic pathway assessment were done utilizing 27 UNinf and 28 VB patients. Phylogenetic analysis revealed that SARS-CoV-2 clades, 19A and 20A, was commonly seen in both unvaccinated (n = 28) and vaccination breakthrough (n = 15) cases, whereas clade 21A was uniquely present in the VB (n = 13).

**Fig 1B** highlights the clinical characteristics of the individual patients who were unvaccinated at the time of SARS-CoV-2 infection and the vaccination breakthroughs. The demographic details of UNinf and VB patients and their hospital captured clinical parameters have been outlined in **Table 1**. The UNinf and VB patients significantly differed (*p ≤ 0*.*01*) in their age. The median age of the UNinf patients was higher 56 years (39–65) whilst that of VB was 36 years (24–56). Compared to the VB, there was a higher preponderance of male patients in the UNinf. All patients from the VB group had received two doses of Covishield COVID 19 vaccine. The time period elapsed after the second dose and breakthrough infection occurrence ranged from ~9 to ~74 days (Avg. 40 days). At the time of hospital admission, SARS-CoV-2 RT-PCR based testing revealed a significantly lower cycle threshold (Ct) value for the *RdRp* in the VB patients (*p ≤ 0*.*01*).

**Table 1:** The demographic details and clinical parameters for unvaccinated and vaccination breakthrough cohort of patients.

	Unvaccinated cases (n = 29)	Vaccination breakthrough cases (n = 29)	*p-value*
Age, median	56 (39–65)	36 (24–56)	**<0.001** ^a^
Gender, F/M	7/22	16/13	0.012 ^b^
Ct value *RdRp* gene	24.21 (22.05–26.45)	17.06 (14.805–22.78)	**<0.001** ^a^
SpO2, median	96 (95–98)	97 (96–98)	0.153 ^a^
Shortness of Breath	9	3	**0.033** ^b^
Respiratory Support	12	0	
Fever	26	19	**0.023** ^b^
Cough	12	16	0.243 ^b^
Sore throat	7	11	0.223 ^b^
Body Ache	4	8	0.274 ^b^
Comorbidity	19	3	**<0.001** ^b^
Hospital stay (Days)	12 (7.5–18)	8 (6–9)	**<0.001** ^a^

Data are shown as median (IQR) or n (%). Statistical significance calculated using ^a^Mann Whitney U test and ^b^Chi^2^ test. Values of significance are highlighted in bold

Mild symptoms of fever, cough, sore throat and body ache were distributed similarly in the UNinf and the VB patients. Although shortness of breath was present in a higher number of patients (n = 9) in the UNinf as compared to the VB (n = 3), yet the partial oxygen pressure (SpO2 levels) measured at the time of hospital admission was within the normal range (» 95–98) for both the groups. A higher proportion of comorbid conditions were also observed in patients from the UNinf (n = 19) than the VB (n = 3), a feature that might alter disease manifestation during the course of hospital stay. Notably, 12 UNinf patients required respiratory support during hospital stay, resulting in a *mild-plus* clinical presentation in the UNinf, whereas none of the VB patients required respiratory support. The hospital stay duration for vaccination breakthrough cases was also found to be significantly lower (*p ≤ 0*.*01*). Taking together, it is evident that both UNinf and VB highlighted mild COVID 19 manifestations but the recovery process was observed to be faster in the vaccination breakthrough patients.

### Dynamic Microbial diversity between unvaccinated and vaccination breakthroughs

**Fig 2:**
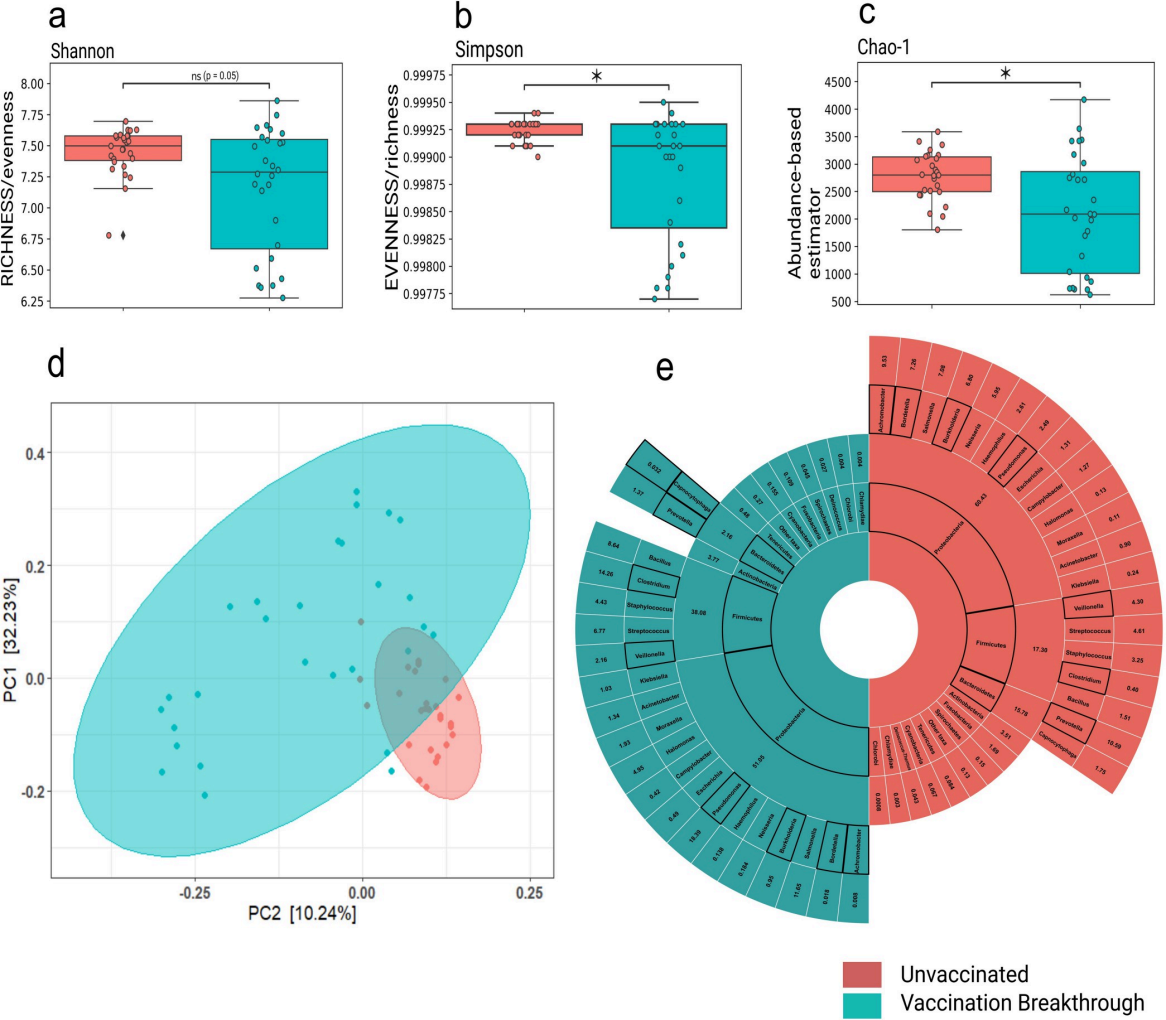
Microbial abundance, richness and evenness distribution across the vaccination breakthrough and the unvaccinated patients. Alpha diversity: a) Shannon (Richness), b) Simpson (Evenness), c) Chao-1 (Abundance-based estimator) showing significant changes in microbiome abundance for vaccination breakthrough and unvaccinated patients, with *p-values* calculated by the Kruskal Wallis test. d) Beta diversity: Principal Coordinate Analysis (PCoA) shows the differential composition of the nasopharyngeal microbes between vaccination breakthrough and the unvaccinated patients. (e) Illustration of the percent differential abundance of phyla and genera across unvaccinated and vaccination breakthrough cases.

With observation towards mild clinical symptoms manifested by both the patient groups’ at the time of hospital admission, yet observing a faster recovery process in the vaccinated individuals, we wanted to investigate the nasopharyngeal microbiome profile associated with the unvaccinated and vaccination breakthroughs. Utilizing the meta-transcriptome analysis pipeline, we identified and analyzed the transcriptionally active microbes to assign taxonomy to the microbial communities. Kraken database detected the co-presence of 5094 transcriptional active microbial species, inclusive of bacteria (96.18% VB & 99.23 UNinf), followed by viruses (3.6% VB & 0.6% UNinf) and archaebacteria (0.1% VB & 0% UNinf). It is important to note that the taxonomic microbial diversity was comparatively higher in the UNinf with Alpha diversity indices, Shannon and Simpson being significantly different between the UNinf and the VB (*p< 0*.*05*) patients (**Fig 2A** and **2B**). Similarly, the abundance based Chao1 values was also found to be significantly (*p< 0*.*05)* higher in the UNinf (**Fig 2C**), suggesting a greater abundance of transcriptionally active diverse microbes in the unvaccinated individuals compared to the vaccination breakthrough. Beta diversity analysis (Bray-Curtis distance matrix) also showed distinct clustering patterns for the UNinf and the VB when visualized in a PCoA plot that explains, 32.3% (PC1) and 10.24% (PC2) of the total variance (**Fig 2D** and **S2 File**). In order to analyse whether the comorbidity observed in the unvaccinated patients is correlated with microbial composition, we partitioned the unvaccinated cohort based on presence and absence of comorbidity and compared the microbial diversity indices. We found that neither Alpha nor Beta diversity showed significance between the groups, negating the role of comorbidity in the observed microbial diversity for the unvaccinated cohort (**S1 Fig**).

Concomitantly, we delved deeper to capture the microbial composition variability at the phylum and genus level (**Fig 2E**). The common abundant phyla observed in both UNinf and VB were *Proteobacteria* (60.4% UNinf vs 51.1% VB) followed by *Firmicutes*, which are natural habitants of the nasopharynx microbiome. Yet, an increase in *Firmicutes* (38.1% VB vs 17.3% UNinf) and decrease in *Bacteroidetes* (2.2% VB vs 15.8% UNinf) phyla abundance was observed between the two groups. The unaltered abundance of *Actinobacteria*, » 3.5% was seen in both the groups whereas that of *Fusobacteria* (0.7% VB vs 1.7% UNinf) differed. Rest of the phyla discovered in our study cohort had less than 1% abundance in the UNinf as well as VB. Looking at the genus level differences, we noticed that the Proteobacteria phyla within UNinf was dominated by several genera, *Achromobacter* (9.5%), *Bordetella* (7.3%), *Salmonella* (7.1%), *Burkholderia* (6.8%), *Neisseria* (6%), *Hemophilus* (2.6%) and *Pseudomonas* (2.5%) whereas VB was overrepresented by only two genus, *Pseudomonas* (18.4%) and *Salmonella* (11%). The genus *Bacillus* of *Firmicutes* showed higher presence in VB (8.6% VB vs 1.5% UNinf) whilst *Prevotella* of *Bacteroides* dominated in the UNinf (10.6% UNinf vs 1.4% VB). **S3 File** contains the specific microbial phyla and genera abundance details which make up the transcriptionally active microbiota of the unvaccinated and vaccination breakthrough patients.

### Microbial features underlying unvaccinated and vaccination breakthrough

**Fig 3:**
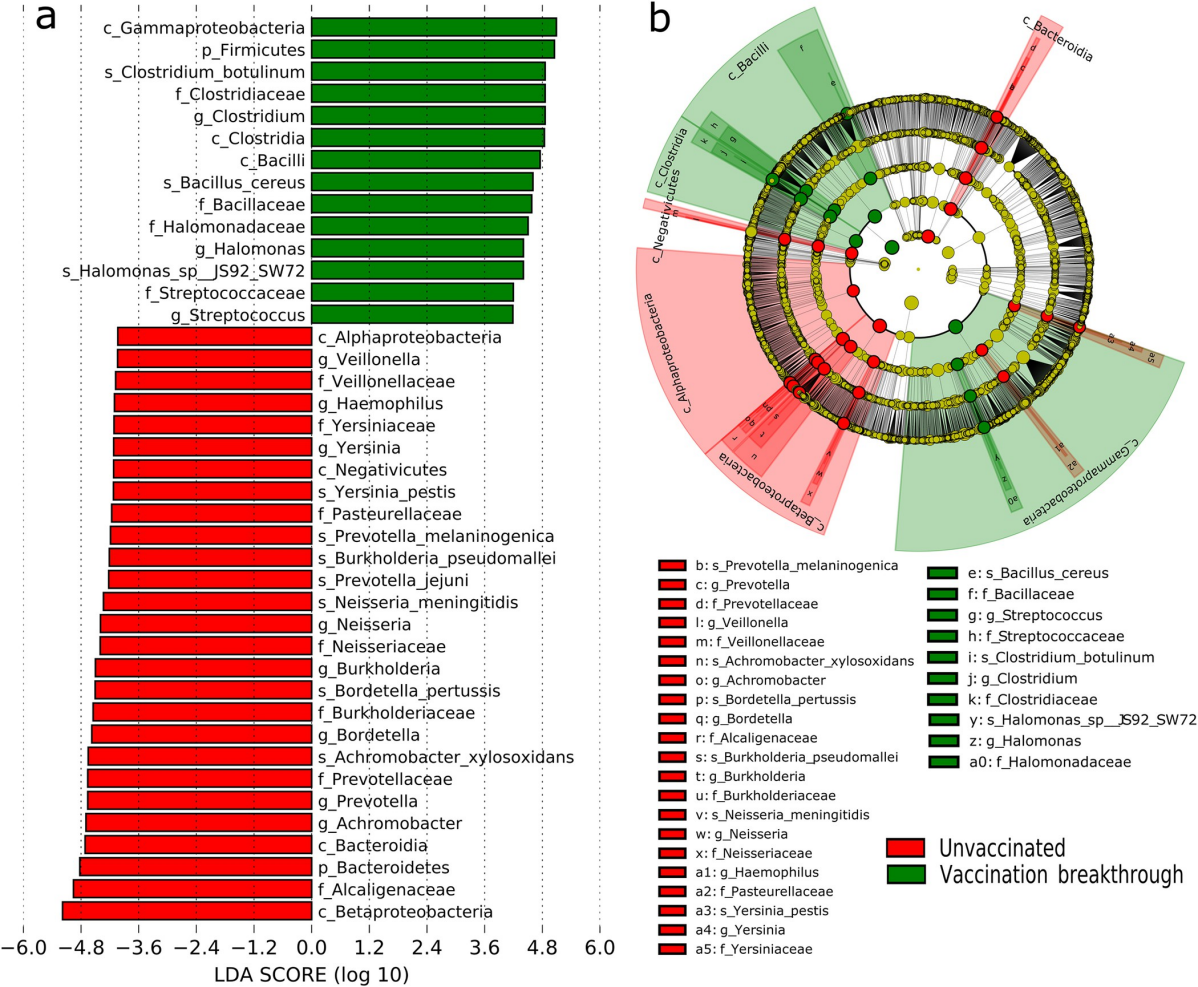
Microbial features across unvaccinated and vaccination breakthrough cohorts. (a) Cladogram (b) Histogram. Differential taxa between two groups selected on the basis of discriminative features by LEfSe analysis (LDA scores >4.0). Green and red indicate taxa enriched in the vaccination breakthrough and the unvaccinated patients, respectively. Depiction of the most abundant microbes at different levels in each group: k, kingdom; p, phylum; f, family; c, class; g, genus; s, species.

The differences observed at the phyla and genus level impelled us to perform linear discriminant analysis (LDA) effect size (LEfSe) to identify the significant taxonomic features that characterize the vaccination breakthrough and the unvaccinated. Using high stringent threshold cut-off >4 for the LDA score, we discovered a notable alteration in the nasopharyngeal active transcription isolates between the two groups. The cladogram illustrates an overlaid microbial composition and prevalence at different taxonomic levels (phylum, class, family, genus and species, denoted by successive circles in the cladogram) for the UNinf and VB with respect to their comparative association (**Fig 3A**). The histogram plot (**Fig 3B**), highlights the identified significantly associated taxon levels that differentiates the UNinf and the VB.

Analysis at the various taxonomic levels showed that phylum *Bacteroidetes* and *Firmicutes* were significantly differentially enriched in the unvaccinated and vaccinated breakthrough patients, respectively. From the phylum *Firmicutes* in the VB, the class domination belonged to *Clostridia* and *Bacilli* with *C*. *botulinum* and *B*. *cereus* species, respectively with significantly distinct abundance. Similarly, for phylum *Bacteroidetes* in the UNinf group, class domination belonged to *Prevotella*, with *P*. *jejuni* and *P*. *melaninogenica* abundance at the species level. The difference in the phylum *Proteobacteria* was evident at the class level where *Alphaproteobacteria* and *Betaproteobacteria* belonged to the unvaccinated cohort with *Gammaproteobacteria* enriched in the vaccination breakthrough. At the species level, *A*. *xylosoxidans*, *B*. *pertussis*, *N*. *meningitidis*, *B*. *Pseudomallei* were found transcendent within the unvaccinated patients whilst several *Pseudomonas* species of *Gammaproteobacteria* aligned with vaccination breakthroughs.

### Differential abundance of opportunistic and commensal microbes between unvaccinated and vaccination breakthroughs

We further analysed the differentially abundant microbial species in the upper respiratory tract of the UNinf and the VB, to ascertain whether the captured microbial features are different/distinct. For stringency and uniformity, the bacterial species with relative cumulative abundance of ≥ 0.1% and presence in at least 70% of the samples within each group, were filtered out. The 70% cut-off was necessitated due to high variance in diversity observed amongst the VB samples. Subsequently, we retrieved 109 bacterial species in the VB group whilst 128 species were identified in the unvaccinated patients. Out of these, 46 and 65 distinct bacterial species were uniquely enriched in the VB and UNinf, respectively. This is in addition to the 63 bacterial species that were commonly abundant in both the aforementioned categories.

**Fig 4:**
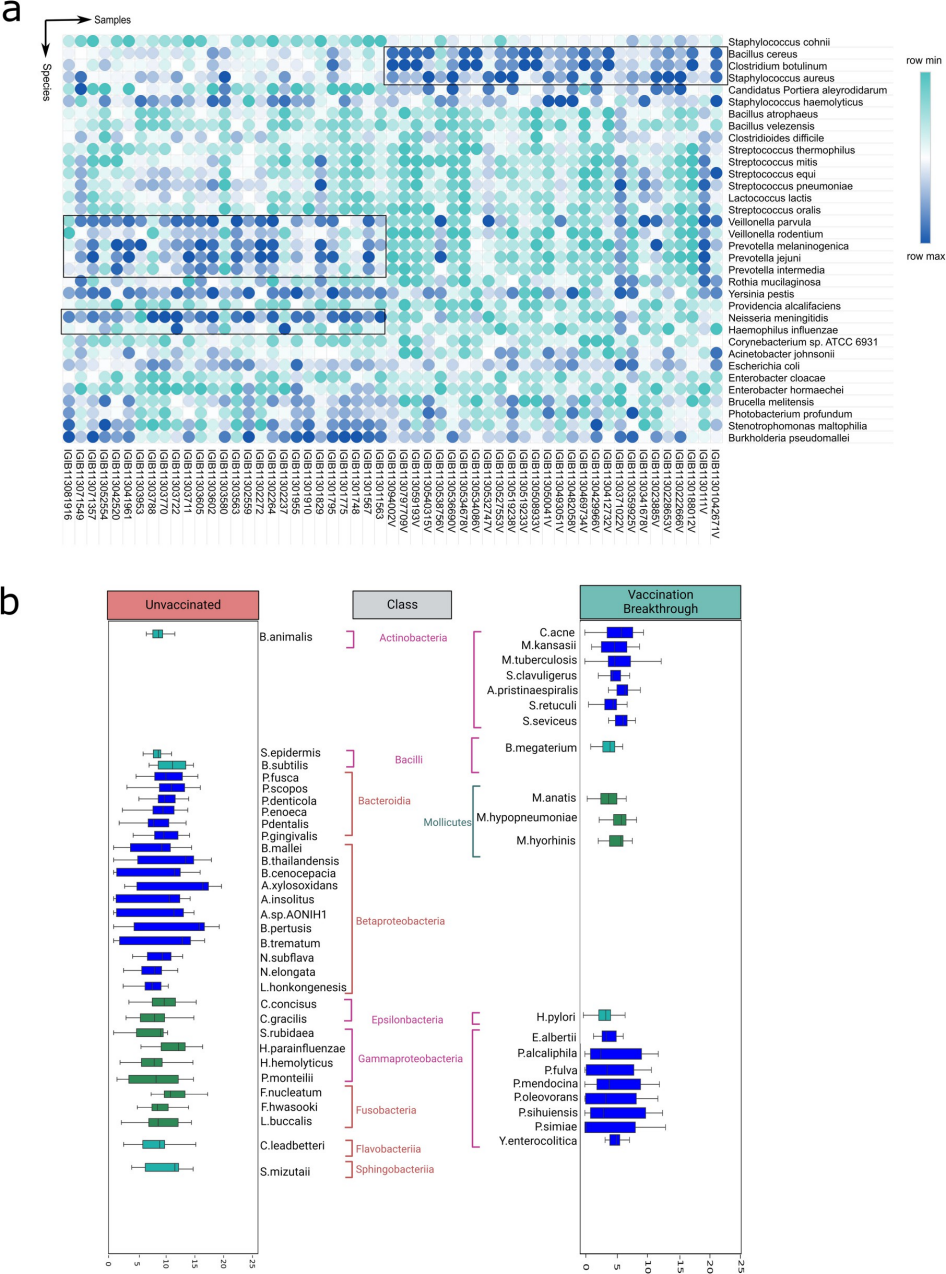
Unique and shared microbial species between the vaccination breakthrough and the unvaccinated patients. (a) Heatmap highlights the sample level dynamics of the common microbial species between the two sub-groups. The sub-clustering of the microbial species has been further highlighted with an encircled box. (b) The microbial class of the differentially abundant unique microbes between the vaccination breakthrough and unvaccinated patients highlight the possible functional dynamics with differential abundance of the commensal and opportunistic microbes.

Firstly, we delved into differential abundance of the common species across the two groups using Mann–Whitney *U* test and obtained 34 significant (*p≤ 0*.*01*) bacterial species, plotted as a heatmap in **Fig 4A**, demonstrating inter-individual species variability across the unvaccinated and vaccination breakthrough. Importantly, we discovered that the opportunistic bacterial species were enriched in the common species with significant higher abundance in the unvaccinated patients. These included *S*. *pneumoniae*, *B*. *pseudomallei*, *N*. *meningitidis*, *R*. *mucilaginosa*, *E*. *coli*, *H influenzae* and *A*. *johnsonii*. The unvaccinated cohort was also populated with diverse species related to genera *Prevotella*, *Veillonella* and *Streptococcus* (*V*. *parvula*, *V*. *rodentium*, *P*. *jejuni*, *P*. *intermedia*, *P*. *melaninogenia*, *S*. *equi*, *S*. *thermophilus*, *S*. *mitis*, *S*. *pneumoniae* and *S*. *oralis*). In contrast, abundance of commensals, such as *S*. *dysgalactiae* and *S*. *salivarius* accompanied by fewer opportunistic species (*B*. *cereus*, *C*. *botulinum*, *S*. *cohnii* and *S*. *aureus*) were found within the VB cohort.

We also explored and illustrated the unique species along with representative class association between the groups (**Fig 4B**). Exclusive presence of class *Betaproteobacteria* enriched with several species of *Achromobacter*, *Burkholderia*, *Bortetella* and *Neisseria* along with *Bacteroidia* (many *Prevotella* species) dominated the unvaccinated individuals; whereas, the enrichers of the vaccination breakthrough included class *Gammproteobacteria* carrying several commensal species of *Pseudomonas* and class *Actinobacteria* inclusive of *Streptomyces*, *Mycobacterium* and *Cutibacterium*. Class *Epsilonbacteria* was present in both the groups with *Campylobacter* in the unvaccinated and *Helicobacter* in the vaccination breakthrough. *Fusobacteria* (*F*. *nucleatum*, *F*. *hawasooki* and *L*. *buccalis*), *Sphingobacteria* and *Flavobacteria* (classes) were only present within the unvaccinated cohort.

### Functional segregation of the differentially abundant active microbial species

We have so far observed a significant difference in the TAMs between the UNinf and the VB, but what possible functional significance could these findings have? Numerous studies show that microbiota can potentially affect vaccination effectiveness and vice versa by modulating the host immune response [12]. Findings highlighting the functional dynamics of the nasopharyngeal microbiota during vaccination breakthrough infections is, however, scarce. In order to understand the underlying mechanism, we delve deeper into the data.

**Fig 5:**
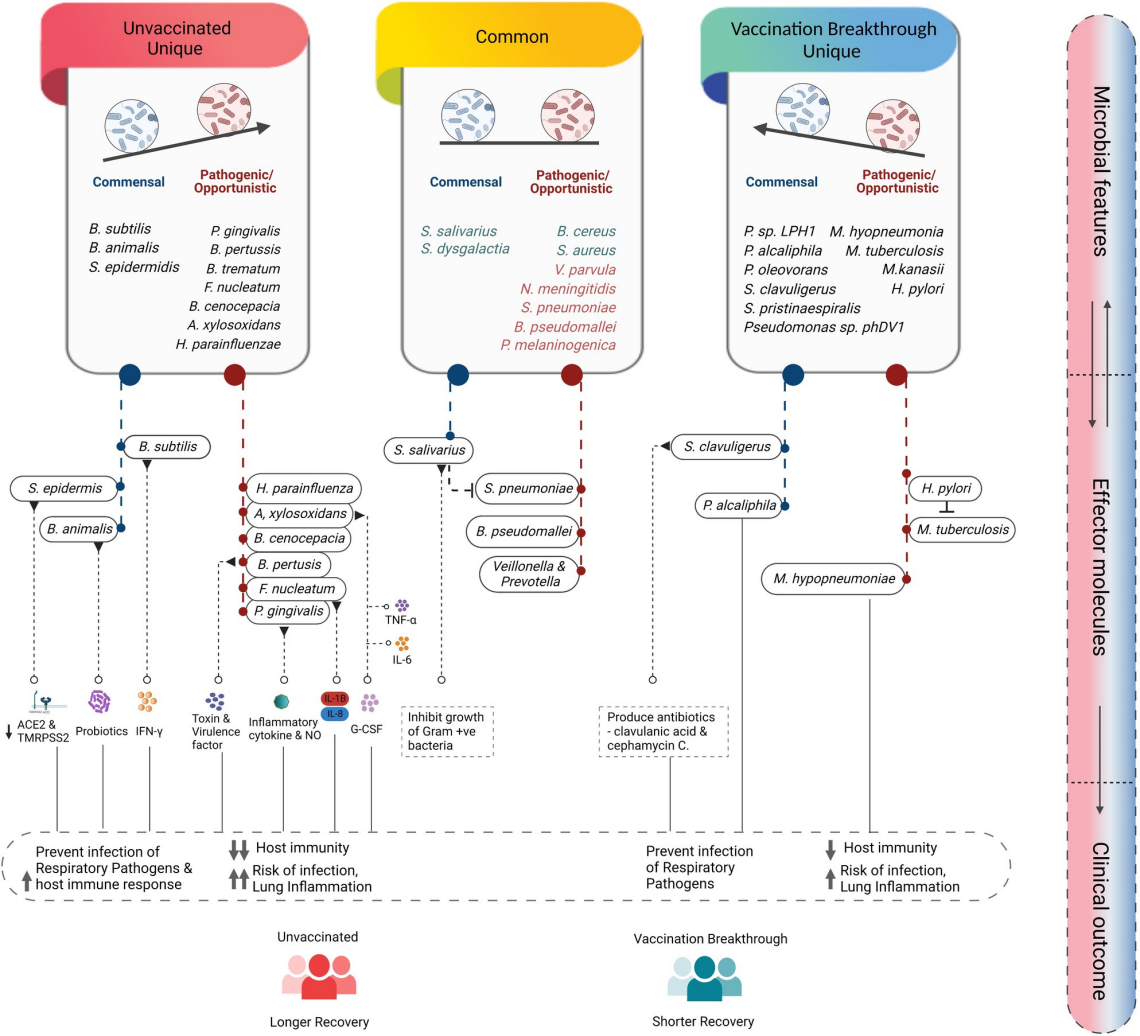
Summary of the differentially abundant microbial species, their known functional role and understanding the disease trajectory. Illustrates the differentially abundant microbial species into commensal and opportunistic, uniquely and shared presence in the groups, functional interpretation, and their role towards distinct immune response in the unvaccinated and vaccination breakthrough individuals correlating with the clinical outcome. Figure created with Biorender.com.

We discovered that the commensal microbes were overridden by opportunistic pathogenic bacteria in the UNinf patients vis-a-vis VB individuals (**Fig 5**). Foremost, we investigated enriched pathogenic bacteria such as *A*. *xylosoxidans*, *B*. *pertussis*, *H*. *parainfluenzae*, *F*. *nucleatum*, *P*. *gingivalis*, and *B*. *cenocepacia* as unique species in the UNinf patients along with *V*. *parvula*, *S*. *pneumoniae*, *B*. *pseudomallei*, and *P*. *melaninogenica* as the common species, found in both the groups. Ample of evidence indicates that the aforementioned species have a role in alleviating disease severity via proinflammatory cytokines (IL-6, TNF-a, G-CSF) release [13] [14] [15] [16]. Therefore, these species might be involved in enhancing lung inflammation that in turn, could be associated with longer recovery rate of the UNinf. At the same time, it is worth noting that, although having a protracted recovery time, the patients remained mild.

This finding piqued our interest, so we dug deeper to find out what was causing UNinf patients’ *mild-plus* clinical profile? In concordance, we observed a few commensal bacterial species including *B*. *subtilis*, *S*. *epidermidis*, and *B*. *animalis*. Studies have identified a fundamental role of these antecedents’ species in boosting host innate immunity via secretion of antimicrobial compounds and IFN-γ [17]. Also, *S*. *epidermidis*, which is a nasal commensal, has been found to downregulate angiotensin-converting enzyme 2 (ACE2) and transmembrane serine protease 2 (TMPRSS2) receptors thereby restricting SARS-CoV-2 entry to the nasal epithelium [18]. Taken together, these aspects may help us to explain the mild plus disease severity and longer hospital stay of the UNinf patients.

On the contrary, we intended to investigate the rationale behind SARS-CoV-2 infection even after two doses of covishield vaccine in the VB patients. The presence of opportunistic species, both unique and common, such as *H*. *pylori*, *M tuberculosis*, *M*. *hyopneumoniae* along with *S*. *aureus*, and *B*. *cereus*, helped us to understand the breakthrough infection. Several studies suggested the contribution of these species in lowering the host immunity and enhancing apoptosis of immune cell malfunction. Consequently, these factors contribute to an increase in respiratory illnesses and the risk of secondary infection. Furthermore, *H*. *pylori*, which usually causes stomach infection, was present in the upper respiratory tract, where it might be altering the outcome of *M*. *tuberculosis* by boosting IFN-γ and Th1-like responses to specific TB antigens, reducing the likelihood of active TB [19].

Additionally, we observed the relatively high preponderance of commensal species in the VB group including *S*. *salivarius*, *S*. *dysgalactiae*, *P*. *alcaliphila*, *S*. *clavuligerus* and *Streptomyces sps*. Studies have revealed the positive impact of the aforementioned commensal species on host immunity. For example, *S*. *salivarius*, an anaerobic bacteria, inhibits the growth of Gram+ve bacteria, particularly *S*. *pneumoniae*, while *P*. *alcaliphila* inhibits the growth of *Legionella pneumophila*, a bacteria that causes human respiratory disease [20] [21]. Collectively, functional characterization of opportunistic/pathogenic and commensal active microbial species aided us to decode the reason behind the clinical phenotype manifested by the UNinf and VB (**Fig 5**).

### Metabolic pathways associated with microbiome diversity strengthens functional role

The microbiome composition and its related functional pathways can potentially alter immune response to infection as well as vaccination [22]. Hence, we elucidated the metabolic pathways that were differentially expressed in the VB and the UNinf along with its association with the bacterial species enriched in each group. Does the differential enrichment of microbial species identified have implications towards immune response and subsequent faster disease recovery in the VB and UNinf? The total metabolic pathways obtained for the microbiota from HUMan3 for the UNinf and VB groups were analysed using the MetaCyc database. The MetaCyc pathways were selectively shortlisted based on the bacterial species that were significantly enriched in the VB and UNinf (common and unique species combined), yielding 95 pathways (**S4 File**).

**Fig 6:**
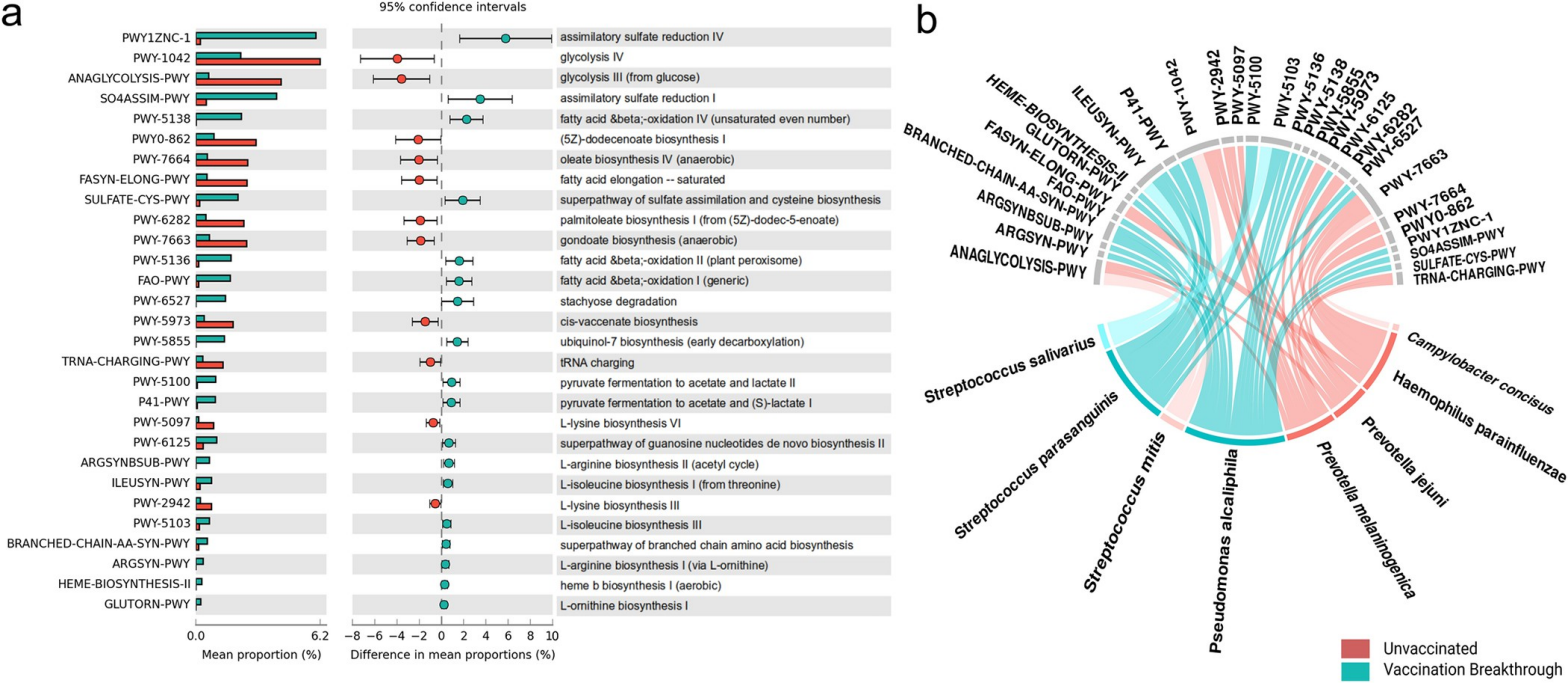
Functional metabolic pathways analysis for the differentially abundant microbial species. (a) Top 29 MetaCyc pathways identified through STAMP analysis which are differentially significantly abundant for the unvaccinated and the vaccination breakthrough patients. (b) Bacterial species contribution from the unvaccinated and vaccination breakthrough cohort leading to enrichment of the top 29 MetaCyc pathways. The color gradient denotes differences in enrichment of pathways based on bacterial species contribution.

STAMP analysis (Statistical Analysis of Metagenomic Profiles) helped us to identify the top 29 significantly enriched metabolic pathways across the two groups (**Fig 6A**). We analysed the difference of each pathway between the UNinf and VB patients and observed that 19 pathways were enriched within VB whilst 10 in the UNinf. Overall, these pathways were associated with carbohydrate metabolism, amino acid biosynthesis and fatty acid oxidation/biosynthesis. For the VB, enrichment was seen for several amino acid biosynthesis pathways of Arginine, Ornithine, Lysine and Isoleucine. It is known that the common human pathogenic bacteria have lost their ability to synthesize amino acids and are completely dependent on the host for the same [23]. Thus, the amino acid biosynthesis pathways signify the dominance of fast-growing commensals rather than potential opportunistic pathogens in the VB. Sulphate assimilatory pathways are significantly upregulated in the VB for the synthesis of cysteine and methionine and a range of other metabolites. Fatty acid and beta oxidation pathways in the VB reveal that high energy producing pathways other than glycolysis are activated in the VB group bacteria whereas glycolysis pathways (anaerobic energy yielding pathway) are significant for the UNinf. Fatty acid and beta oxidation pathways also lead to production of unsaturated shorter chain fatty acids, which might contribute towards immune response generation. Contrarily, the enrichment of a large number of long chain fatty acids (LCFAs) biosynthesis pathways—dodecenoate biosynthesis I, oleate biosynthesis IV (anaerobic), palmitoleate biosynthesis I (from dodecenoate), cis-vaccenate biosynthesis and gondoate biosynthesis in the UNinf cohort, may lead to the generation of LCFA molecules, known to play a role in inflammation [24].

The analysis of species contribution in the differentially enriched pathways using HUMAnN3 led to the identification of eight bacteria responsible for major metabolic activities in the unvaccinated (**Fig 6B**) and vaccination breakthrough patients (**Fig 6C**). *P*. *jejuni* and *P*. *melaninogenica* were the metabolically dominant species in the unvaccinated, showing highest association with all the glycolysis pathways, followed by cis-vaccenate and gondoate biosynthesis pathways for the LCFA generation. tRNA charging and Lysine biosynthesis pathway were uniquely observed for *P*. *jejuni* and *P*. *melaninogenica*. Notably, all of the LCFAs biosynthesis pathways mentioned above were mainly attributed to *H*. *parainfluenze*, an opportunistic pathogen present in the unvaccinated. *S*. *mitis* and *C*. *concisus* were the other bacterial species which contributed to glycolysis pathway and gondoate (LCFA) biosynthesis from the unvaccinated cohort. In the vaccination breakthrough, highest enrichment was seen for *P*. *alcaliphila*, which was responsible for all the significant metabolic pathways captured. *S*. *salivarius* and *S*. *parasanguinis* also contributed to isoleucine biosynthesis pathway along with *P alcaliphila*, whereas *S parasanguinis* was uniquely responsible for the enrichment of pyruvate fermentation to acetate and lactate pathway, important metabolites for the SCFA generation.

### Functional concordance of disease severity associated microbial species across cohorts

**Fig 7:**
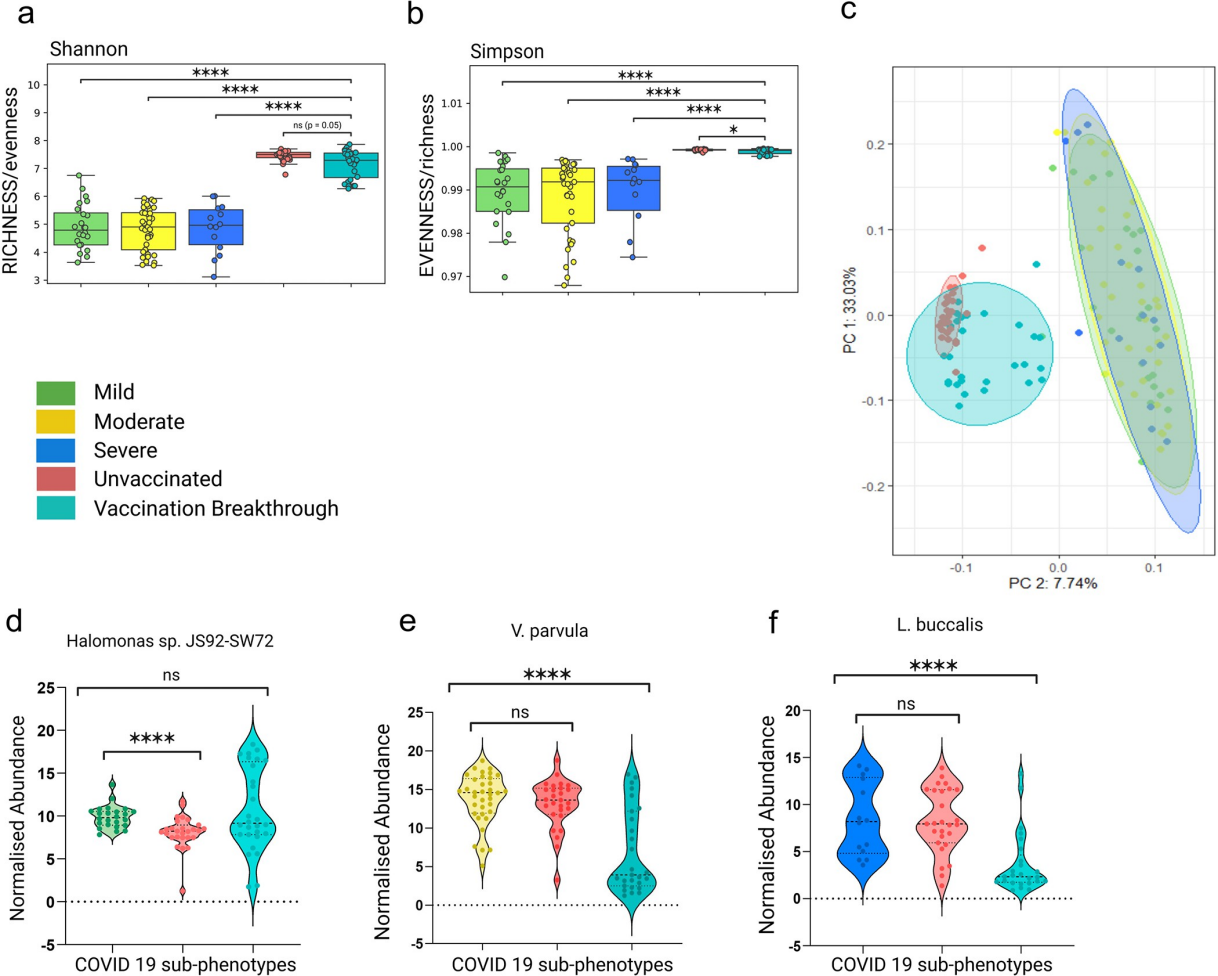
Comparison of microbial diversity between the two cohorts and validation of the disease severity associated microbial species. Alpha diversity comparison (a) Shannon (b) Simpson of COVID 19 sub-phenotypes (mild moderate & severe) with unvaccinated and vaccination breakthrough groups. (c) Principal Coordinate Analysis (PCoA) plot representing the beta diversity of bacterial species across mild, moderate, severe, unvaccinated and vaccination breakthrough groups. (d-f) Comparison of three differentially abundant bacterial species (*Halomonas sp*) in mild, (*V*. *Parvula*) in moderate and (*L*. *Buccalis*) in severe with unvaccinated and vaccination breakthrough cases, respectively.

For broader relevance/specific importance of the findings presented here, we compared the current microbial profiles to those of COVID 19 infected patients from an earlier published work from the lab with differential disease severity of mild, moderate, and severe. Compared to the UNinf and VB cohort, the alpha-diversity indices, both Shannon and Simpson, were significantly lower in the COVID 19 severity groups (**Fig 7A and 7B**). Similarly, the previous cohort of mild/moderate/severe patients yielded a significant degree of separation from the UNinf/VB with respect to beta diversity principal coordinate analysis (PCoA) (Bray-Curtis distances). This highlights that the two-time separated, different patient cohorts had distinct microbial compositions (**Fig 7C**). But it is important to note that although the microbial diversity is dynamic across these two cohorts, the functional importance of the findings in terms of the microbes associated with the disease severity, from the earlier study, is validated in the present cohort. The transcriptionally active microbial species, *Halomonas sp*. (mild), *Veillonella parvula* (moderate) and *Leptotrichia buccalis* (severe) were queried for their abundance in our UNinf/VB dataset across patients.

The *Halomonas sp*. that exhibited correlation with the mild group showed substantial difference with the UNinf group (*p < 0*.*01)*. This is possibly owing to the presence of a higher number of patients with shortness of breath in the UNinf group, compared to the VB cases (p = 0.59) with no respiratory symptoms (**Fig 7D**). *Veillonella parvula*, discovered to be enriched in the moderate group (symptoms of breathing difficulty with SpO_2_ levels between 91–93%) differed significantly from the VB group *(p < 0*.*01)*, revealing further that it is linked to a relatively severe symptoms as compared to the mild condition (**Fig 7E**). Like how *Leptotrichia buccalis* was linked to the severe group in the prior cohort, it was associated with the comparatively severe group of this cohort i.e., UNinf, whereas no discernible difference from the unvaccinated group *(p = 0*.*83)*. Importantly, it showed more pronounced discrepancies within the vaccination breakthrough group with milder presentation *(p = 0*.*0001)* (**Fig 7F**). Thus, it helps to highlight that the microbial species that we discovered in our earlier research are pertinent in this instance with relative difference in disease severity (difference in the hospital stay period). This corroborates the conclusions of earlier study’s identification of the severity linked TAMs in the COVID 19 sub-phenotypes.

## Discussion

Vaccines have played a crucial role in combating, containing and eliminating many serious diseases, including measles, mumps, tetanus, pneumococcal and the influenza type B. The immune response to vaccination varies from person to person, despite it being a very effective and successful strategy. Since the launch of vaccination for the COVID 19 pandemic, vaccination breakthrough cases have continuously challenged the vaccines’ effectiveness and provided incentives/leads to explore the facets which holds potential to alter vaccination induced immunity and protection from subsequent infection. The human/nasopharyngeal microbiota is one such facet. To the best of our knowledge, this is the first study which has investigated the vaccination breakthrough cases for its association with the upper respiratory tract microbes in the context of unvaccinated SARS-CoV-2 infected cases.

Growing evidence suggests that microbes and their molecules influence the immune response to vaccination against viruses such as influenza and SARS-CoV-2 [25] [26]. This phenomenon is evidently observed in our study where significant differences in the diversity of upper respiratory tract microbes was witnessed between the vaccination breakthrough and the unvaccinated patients. We discovered that the vaccinated group had relatively less diverse microbiota with specific prominent taxonomic communities dominating the microflora (**Fig 2E**). Contrarily, the unvaccinated SARS-CoV-2 infected individuals harboured a greater diversity of microbiota at all taxonomic levels. Although, it seems plausible that a well-balanced high diversity microbiota fosters a more protective immune response to vaccines [27] yet, we can deliberate that a shift in the diversity due to vaccination leading to enrichment of particular bacterial communities that substantiates immune response generation, can be beneficial during an event of re-infection leading to reduced clinical manifestations.

Studies have reported that altering the microbiota composition to increase the presence of *Gammaprotiobacteria* and reducing *Bacteroidetes* generates favourable immune response post vaccination with rotavirus vaccine [28] [8]. In line with this finding, the VB group of our study also demonstrated enrichment of *Firmicutes* and *Gammaproteobacteria* (phylum Proteobacteria) with highly reduced *Bacteroidetes* when compared with UNinf individuals. Contrarily, the UNinf microbiota highlighted *Bacteroidetes* abundance governed by *Prevotella* which is consistent as core anaerobic bacterial communities of the respiratory tract [29]. Although commensals, they have immune-modulatory activity and alteration in species richness to high densities can lead to low-dose inflammation and mild to severe respiratory disease conditions [30]. Additionally, an upheaval of potential opportunistic microbes belonging to *Proteobacteria*, with *Neisseria* and *Bordetella* of *Betaproteobacteria*, while, *Hemophilus* from *Gammaproteobacteria*, *Burkholderia* and *Achromobacter* from *Epsilonproteobacteria* enriched the pathogenic potential of the unvaccinated patients’ microbiota [31].

It is imperative to state that the microbes which induce protective responses in the host (commensals) and that take advantage of a host with a weak immune system to negatively impact the host (opportunistic/pathogenic) tend to influence human fitness, and can potentially affect the individual’s response to vaccination induced protection. Similarly, the microbial communities that were altered in the background of immunization in our study demonstrated differential dynamic persistence of commensals in vaccination breakthrough cases as compared to opportunistic pathogens in the non-immunized SARS-CoV-2 infected group.

The unique presence of a high number of opportunistic pathogens such as *A*. *xylosoxidans*, *B*. *pertussis*, *H*. *parainfluenzae*, *F*. *nucleatum*, *P*. *gingivalis*, and *B*. *cenocepacia* along with several significantly abundant common potential pathogens, *V*. *parvula*, *S*. *pneumoniae*, *S*. *mitis*, and *P*. *melaninogenica* of UNinf cohort can appreciably reduce host immunity causing higher risk of infection and lung inflammation (**Fig 5**). These detrimental microbial features are in concordance with the overrepresentation of metabolic pathways of LCFAs biosynthesis in the UNinf group since all LCFA molecules (oleate, dodecenoate, palmitoleate, as well as gondoate), have been shown to have an effect on the level of inflammation in the lung of cystic fibrosis patients [24]. Among the major potential opportunistic pathogens of the UNinf group, *H*. *parainfluenze*, known to cause respiratory tract infections, dominated the LCFAs generation. LCFAs provide metabolic energy to the pathogenic bacteria and fitness advantage to human pathogens like *Vibrio cholera* [32]. The other metabolically dominant species of the unvaccinated cohort, *P*. *melaninogenica* and *P*. *jejuni*, showing highest association with glycolysis pathways and LCFA generation, are reported to be highly abundant in the COVID 19 patients. Given the association of LCFAs with persistent presence of opportunistic pathogens, the mild-plus presentation of COVID 19 infection can be explicated for the UNinf patients. However, the co-presence of a few commensal species, including *B*. *subtilis*, *S*. *epidermidis*, and *B*. *animalis*, that are known to secrete antimicrobial compounds and IFN-γ [17][33], might operate as a host shield, subsequently improving host immunity to fight infection thereby ameliorating severe disease conditions.

The microbiota of vaccination breakthrough cases exhibited distinct beneficial features with relatively higher abundance of unique commensal organisms. The presence of several species of *Streptomyces*, uniquely abundant in the VB individuals, are source of bioactive secondary metabolites, such as antifungals, antivirals, anti-hypertensives, immunosuppressants, and especially antibiotics [34]. They may potentially alter the behaviour of the microbiota and pathogens through specific antibiotic activity or quorum quenching mechanisms [35]. *S*. *clavuligerus* produces clavulanic acid, an irreversible β-lactamase enzyme inhibitor, used to treat variety of clinical syndromes including pneumonia and exacerbated chronic obstructive pulmonary disease (COPD) [36].

The other commensal species such as *S*. *salivarius*, *S*. *dysgalactiae*, and *P*. *alcaliphila* exert their beneficial effect by inhibiting the growth of other human respiratory pathogens such as *S*. *pneumoniae* and *L*. *pneumophila*, respectively [37] [38]. Additionally, various strains of *S*. *salivarius*, a prominent member of the healthy oral microbiota, has been reported as an excellent bacterial probiotic limiting the detrimental effects of all strains of *S*. *pyrogenes*, a common cause of pharyngeal infections. The significant protective effect that *S*. *salivarius* exhibits to potential pathogens, might have health benefits by contributing towards milder disease in the VB patients. Another study has elucidated the bioactive potential of *P*. *alcaliphila* against several other human bacterial pathogens such as *Klebsiella pneumoniae*, *Salmonella Typhi*, *E*. *coli*, and *Bacillus subtilis* [21].

**Fig 8:**
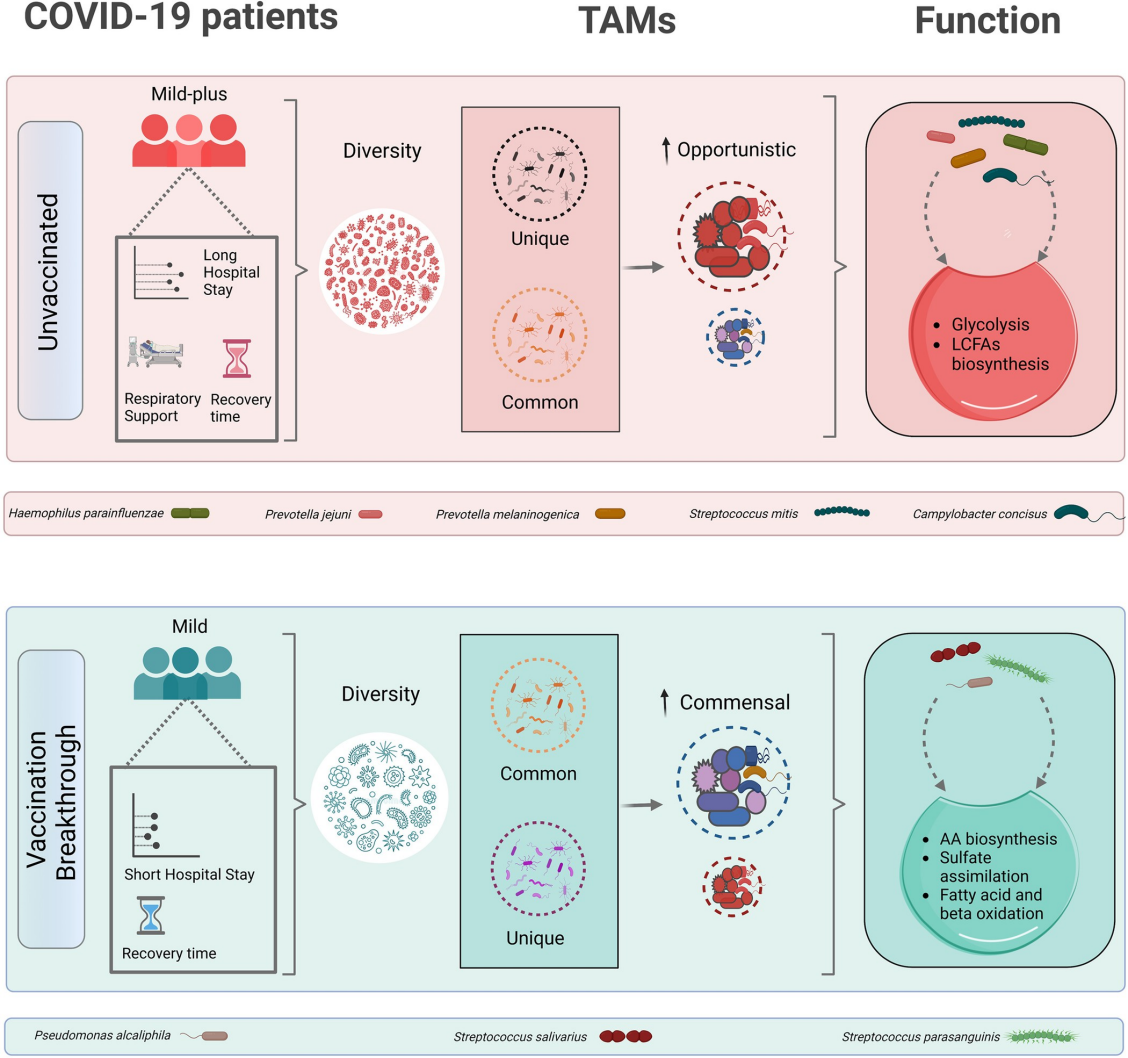
Summarizes differential abundance of commensals and opportunistic microbes modulating disease trajectory in vaccination breakthroughs and unvaccinated infections of SARS-CoV-2. Figure created with Biorender.com.

Interestingly, *P*. *alcaliphila* seems to be one of the potential commensals dominating the pseudomonas genera (*Gammaproteobacteria*) distinctively abundant in the VB cohort. As explained above, *Gammaproteobacteria* have been reported to be beneficial for favourable immune response post vaccination. This response can be implicated to the commensal pseudomonas and in the present case, *P*. *alcaliphila*. Moreover, further investigation of metabolic functional pathways, along with the species contribution to the differentially enriched pathways, corroborated that the VB group had enrichment of pathways related to amino acid biosynthesis, sulphate assimilation, fatty acid and beta oxidation, all of which were positively correlated with abundance of *P*. *alcaliphila*. Findings have indicated that fatty acid beta oxidation pathways and amino acids biosynthesis may be utilized to synthesize acetate, butyric acid, and propionic acid, all of which are SCFAs known to guide immune responses positively and also provide resistance to pathogenic microbes [39]. Collectively, these findings implicate that vaccine induced shift in microbiota towards enrichment of commensals correlate with milder symptoms and a shorter hospital stay as observed in the vaccination breakthrough patients (**Fig 8**).

Further study in this direction towards understanding limited, yet important, subset of severe vaccination breakthrough infections as well as role of emerging variants would be useful. In addition to COVID-19, similar studies for other vaccinations (like Flu) would be useful to understand the dynamics between post-vaccination states of prevention of infection and mild symptoms in vaccination breakthrough.

## Conclusions

Our study demonstrates alteration in the upper respiratory tract microbial profile at different taxonomic levels of individuals belonging to SARS-CoV-2 infected UNinf and VB at the time of hospital admittance. In the context of vaccination, how the nasal microbiome is altering and responding to vaccination? What led patients to manifest similar clinical presentation, mild, but longer hospital stay in the unvaccinated ones? Answers to some of these questions is provided by the directional perturbation in the abundance of residing commensal and opportunistic pathogens of nasopharynx. Corroboration with the functional analysis highlights their involvement in the pathogenesis via modulating the immune response. Furthermore, current study reveals the enrichment of microbial derived metabolic pathways, as well as their association with bacteria in the respective groups. As a result, this research aided us to comprehend the plausible mechanism underlying the variable impact of vaccination at the population level. Last but not the least, it is important to note that although the microbial diversity in the time separated cohorts are different, the discovery with respect to disease severity associated microbial species, was further validated by their differential abundance between the unvaccinated and vaccination breakthrough. This was in alignment with the relative disease severity differences between the sub-groups being investigated.

## Materials and methods

### Ethics statement

The studies involving human participants were reviewed and approved by the Council of Scientific and Industrial Research- Institute of Genomics and Integrative Biology’s (CSIR-IGIB) Human Ethics Committee Clearance (Ref No: CSIR-IGIB/IHEC/2020-21/01). The patients/participants provided their written informed consent to participate in this study.

### Sample collection and study design

Nasopharyngeal swab samples of hospital admitted COVID 19 patients were collected in Viral Transport Medium (VTM) (HiViral Transport Kit, HiMedia, Cat. No: MS2760A-50NO) at the Max Hospital, Delhi as per the standard guidelines of Indian Council of Medical Research (ICMR). The research was carried out by the CSIR-Institute of Genomics and Integrative Biology (CSIR-IGIB) in collaboration with Max Hospital. Ethical clearance for the study was obtained from the Institutional Ethics Committee at the CSIR-IGIB and the Max Hospital, respectively. The samples were blindfolded with double barcoding to avoid tracing back the findings made in the study at the individual patient level. 3ml VTM tube containing swab samples was vortexed & centrifuged for proper dissolution and settlement of samples before downstream processing. Alongside, the detailed hospital registered clinical data of the patients was also recorded electronically. Patients were categorized into two groups: i) Unvaccinated & infected (UNinf), and ii) Vaccination breakthrough (VB), to explore the transcriptionally active nasopharyngeal microbiota and its association with vaccination status when compared to unvaccinated individuals, with both cohorts being SARS-CoV-2 infected at the time of the study.

### Viral isolation and qRT-PCR

For viral RNA isolation, 150 ul sample was used and extracted using QIAmp viral mini kit, Qiagen, Cat. No. 52906. Isolated RNA was used for RT-PCR using TRUPCR SARS-CoV-2 kit (3B BlackBio Biotech India Ltd., Cat. No. 3B304) with a cycle threshold of 35 to detect and quantify the SARS-CoV-2 infection.

### Whole genome sequencing of SARS-CoV-2

Genome sequencing was performed according to Oxford Nanopore Technology (ONT) library preparation protocol (Version: PCTR_9125_v110_revB_24Mar2021). 50ng of total RNA was used to synthesize single stranded cDNA using LunaScript RT SuperMix (New England Biolabs, Cat. No.E3010L). The cDNA-RNA hybrid was used to amplify SARS-CoV-2 genome with rapid barcoding primers (IDT Product number: 10007184) and Q5 High-Fidelity 2X master mix (New England Biolabs, Cat. No. M0494S). For sequencing, the amplified products were ligated with rapid barcode sequences (SQK-RBK110.96) followed by SPRI bead purification. The purified library was then ligated with adapter protein and loaded on the MinION Mk1C platform. The ARTIC end-to-end pipeline was used for the analysis of ONT MinION raw fast5 files up to variant calling. The resultant demultiplexed fastq were normalised by read length of 300–500 for further downstream analysis and aligned to the SARS-CoV-2 reference (MN908947.3) using the aligner Minimap2 v2.17 [40]. Nanopolish was used to index raw fast5 files for variant calling from the minimap output files [41]. To create consensus fasta, bcftools v1.8 was used with normalised minimap2 output. SARS-CoV-2 genomes (with >50% genome coverage) were used for phylogenetic analysis. Clade assignment to all the genomes was done using Nextclade (https://clades.nextstrain.org/).

### Dual RNA-Sequencing

Transcriptome sequencing was performed to probe the presence of transcriptionally active microbes using the Illumina TrueSeq Stranded Total RNA Library Prep Gold (Illumina, Cat. No. 20020598). Before proceeding with cDNA synthesis for downstream library preparation, the depletion of cytoplasmic rRNA and bacterial rRNA was carried out with 250 ng input total RNA from each sample. Following cDNA synthesis, purification of double stranded cDNA was carried out with AMPure XP beads (AMPure XP, Beckman Coulter, Cat. No. A63881) in accordance with the reference guide (Illumina, Doc. No. 1000000040499 v00). Library preparation included adenylation at 3′ends and ligation with index adapters. Subsequently, PCR based amplification was performed to enrich the cDNA libraries. PCR products were purified using AMPure XP beads and quantified using Qubit dsDNA HS Assay kit (Thermo Fisher Scientific, Cat. No. Q32854). The quality of cDNA libraries was checked by Agilent High Sensitivity DNA Kit using the Agilent 2100 Bioanalyzer. A final loading concentration of 650 pM was prepared by diluting the libraries and sequencing was performed on the NextSeq 2000, and paired end 2×151 read length.

## Metatranscriptomic analysis

### Sequence data processing

Sequencing Raw reads were quality filtered and trimmed using Trimmomatic v0.39 to remove adaptor and low-quality sequences. It was then aligned with HISAT2 to remove human host RNA reads by mapping reads onto human reference genome GRCh38. Samtools was used to filter human unaligned reads for downstream analysis to identify the transcriptionally active microbes.

### Functionally active microbial mapping and taxonomic classification

Kraken2, a taxonomic classifier that maps shotgun sequencing k-mers to genomic databases, was used to assign taxonomy on filtered and pre-processed reads. The database was downloaded which is built from the refseq bacteria, archaea and viral libraries. The kraken2 function was used to run the filtered reads against this database and assign taxonomy. While Kraken2 does not estimate species abundances, Bracken2 (Bayesian Reestimation of Abundance with KrakEN) uses the taxonomy assigned by Kraken2 to estimate the number of reads per sample that originate from the individual species. The Kraken2 database was used to create a Bracken-compatible database using the brackenbuild function, and the Kraken2 report files for each sample were run against the Bracken database using the bracken function for the phylum, genus and the species level information.

Phylum- and species-level relative abundance outputs were formatted for biomarker discovery using LEfSe [42]. The kraken-biom function was used to convert the Bracken report files into a biom file for import into R. CSS normalization was used to normalize the read count generated from the Bracken [43]. Taxonomic Diversity Analysis through Alpha and Beta diversity were performed using the phyloseq (v1.27.2) [44] and vegan (v2.5–4) (Dixon, 2009) packages in R (v3.4.3). Towards this, the biom file was imported into a phyloseq object. Alpha diversity (within-sample diversity) indices, including Shannon, Simpson and Chao-1 index, were calculated on the basis of the species profile for each sample. Beta diversity (between-sample diversity) was calculated as bray-curtis index by the phyloseq and vegan packages and visualized by Principal Coordinate Analysis (PCoA) plot. Data was exported as csv files and formatting, plotting and visualization was performed in python (seaborn library).

### Metabolic Pathways analysis and bacterial species contribution for the microbiome

The Human Microbiome Project Unified Metabolic Analysis Network 3 (HUMAnN3) pipeline was used to profile the presence and abundance dynamics of metabolic pathways in the nasopharyngeal microbiota of our patient cohorts [45]. Briefly, the HUMAnN function was used with the filtered metagenomic sequences to estimate pathways present in the samples based on the UniRef90 database. Files were normalized from abundance RPK to CPM using humann_renorm_table and then joined into one table using the humann2_join_tables function. These relative abundance tables were formatted for statistical significant analysis with STAMP [46]. Following this, microbiota taxonomic compositions and functional pathways were inferred from filtered reads using HUMAnN (v3.0) and STAMP (v2.1.0), respectively, with default settings.

### Statistical analysis

The clinical data was presented as descriptive statistics, which displays continuous variables as medians or interquartile ranges and categorical variables as percentages or proportions. Wherever appropriate, we compared the significance between the groups using Mann–Whitney *U* test and Chi square test. The distribution of bacterial presence across unvaccinated and vaccination breakthrough patients was calculated using Kruskal Wallis test. For Beta diversity analysis, PERMANOVA was calculated to determine the statistical significance (https://palaeoelectronica.org/2001_1/past/issue1_01.htm)). Statistical significance for different metabolic pathways were screened by STAMP for the unvaccinated and vaccination breakthrough cohorts. Fig 1A (publication license no. WP24XFCCDO), Fig 1B (publication license no. QJ24XFC0KJ), Fig 5 (publication license no. NP24XFBAJZ) and Fig 8 (publication license no. LD24XFAMQK) were created with BioRender.com.

## Supporting information

S1 File Distribution of microbial reads for all samples from the RNA-Seq data set. (XLSX)

S2 File Alpha diversity indices showing bacterial species richness, evenness and abundance across the patients in unvaccinated and vaccination breakthrough cohort. (XLSX)

S3 File Taxonomic communities at the level of phylum and genus, identified through Bracken in unvaccinated and vaccination breakthrough cohort. (XLSX)

S4 File Total MetaCyc pathways identified through STAMP analysis across the unvaccinated and vaccinated breakthrough cohort with statistical *p*-adjusted value. (XLSX)

S1 Fig Microbial abundance, richness and evenness distribution across the comorbid and non-comorbid unvaccinated patients. (JPG)
