# Supplementary figures and images for: Transcriptionally active nasopharyngeal commensals and opportunistic microbial dynamics define mild symptoms in the COVID 19 vaccination breakthroughs
Source: PLoS Pathog. 2023 Feb 17;19(2):e1011160. doi: 10.1371/journal.ppat.1011160 (PMC9937460; doi:10.1371/journal.ppat.1011160)

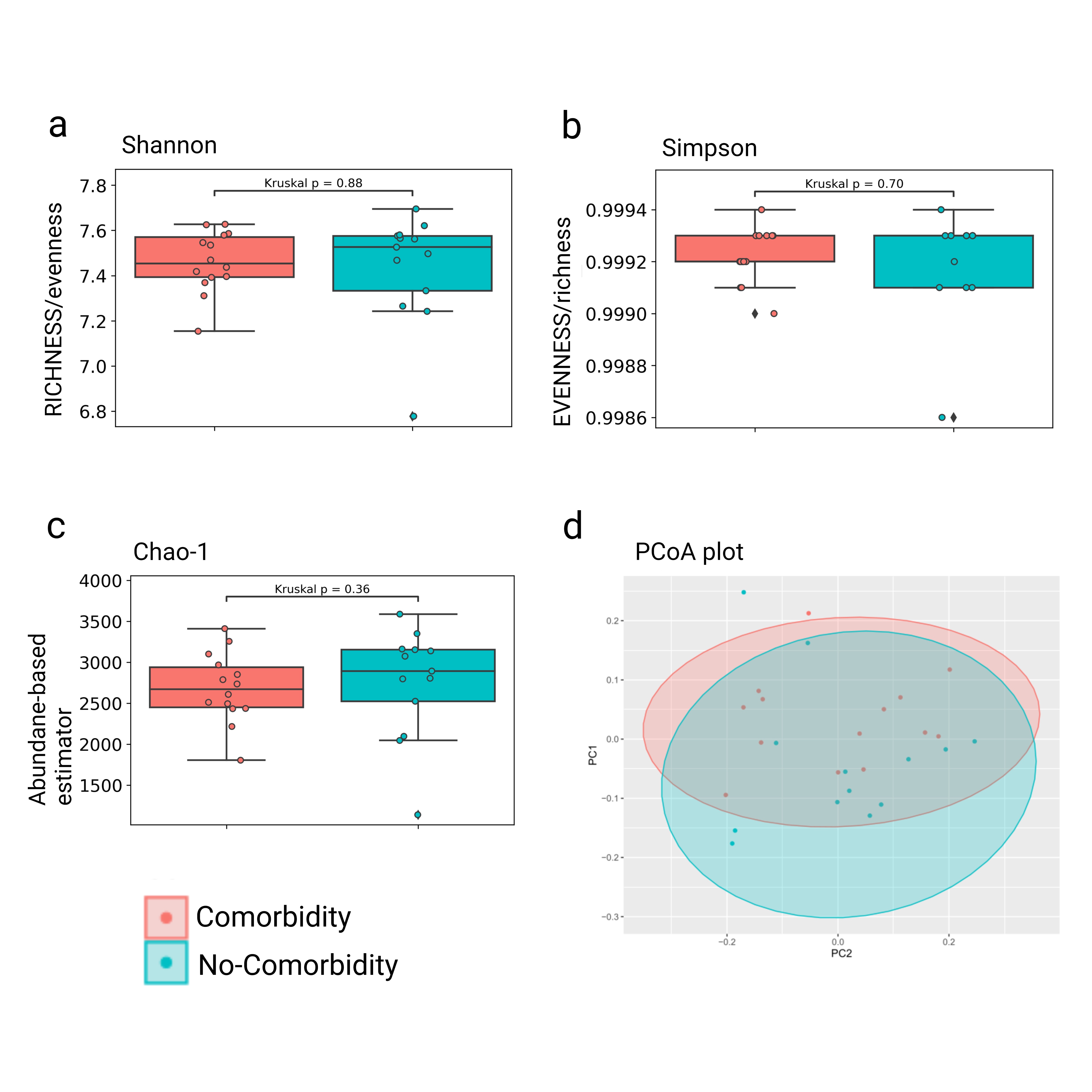

Supplement: S1 Fig — (JPG) [file ppat.1011160.s005.jpg]
